# A triple-layer authentication framework with elliptic curve cryptography for securing IoT-assisted wireless sensor networks

**DOI:** 10.1371/journal.pone.0329011

**Published:** 2025-08-13

**Authors:** Meshari D. Alanazi

**Affiliations:** Department of Electrical Engineering, College of Engineering, Jouf University, Sakakah, Saudi Arabia; Jaramogi Oginga Odinga University of Science and Technology, KENYA

## Abstract

Wireless Sensor Networks (WSNs) are integral to the Internet of Things (IoT), yet they face significant security challenges, including vulnerabilities to various attacks like Man-in-the-Middle and insider threats. Traditional security mechanisms are often insufficient in providing the necessary level of security while accommodating the processing and energy limitations of resource-constrained devices. Hence the research proposes an innovative Elliptic Curve Cryptography and Triple-Layer Authentication Framework (ECC-TLAF) that enhances the security of WSNs while improving energy efficiency and reducing latency. The framework utilizes a lightweight authentication approach involving biometrics, smart cards, and passwords. The research results indicate that the ECC-TLAF model significantly enhances performance, achieving a 98.9% improvement in energy efficiency, a 95.6% increase in security, and a 96.7% attack detection rate. Moreover, it reduces processing time by 1.02 seconds and latency by 0.1 milliseconds compared to conventional frameworks. The ECC-TLAF framework effectively addresses the security challenges in WSNs, ensuring robust protection against cyber threats while optimizing resource utilization. This research contributes to the advancement of security protocols for IoT environments, paving the way for more secure and efficient WSN applications.

## 1. Introduction

The extensive implementation of wireless sensor networks (WSNs) has made communication security a key research focus [1]. This is because WSNs are used almost everywhere. Keeping data secret while preventing attackers from obtaining it is a significant challenge on the WSN enabled by the IoT [2]. A great number of authentication and key agreement security concerns are present in the techniques that are currently in use. They fail to fulfil anonymity and forward security criteria because of their susceptibility to attacks like MITM (Man-in-the-Middle), insider privileges, password guessing, hiding users, and other similar techniques [3]. Building user authentication methods using session keys is a common method for addressing the issues mentioned above with IoT-enabled WSNs [4]. The traditional design of a WSN includes nodes for users, gateways, and sensors. In general, gateway nodes are unrestricted in their memory, processing power, and ability to maintain the integrity of their long-term keys [5]. Instead, sensor nodes function in unsupervised contexts and are often small, inadequate, and attackable. These sensors detect and record various physical and environmental factors, including vibration, temperature, pressure, and sound [6]. The gateway mediates communication between the sensor nodes and the users, permitting them to access real-time data after authorization [7]. In a typical setup, the user transmits a data request to gateway nodes, storing the data sent by sensor nodes to be retrieved by the user. On the other hand, users often need to get real-time data straight from sensor nodes when there’s a big demand, such as detecting enemies or getting situational awareness on the battlefield [8]. A hacker can only compromise the protocol’s security by compromising all authentication elements used in the networking applied in banking, finance, IoT and other sectors; compromising only one will have no effect. The symmetric key approach is the core of authentication and identifying the network users sequential pattern helps to overcome the resource limitations of sensors [9].

Strong elliptic curve cryptography (ECC) can protect wireless sensor networks (WSNs) [10]. WSNs are found in many places and devices. Security vulnerabilities to WSN sensor nodes include eavesdropping, data manipulation, and node capture [11]. Small size limits memory, computer power, and battery life. In wireless sensor networks (WSNs), RSA and other encryption techniques are inadequate [12]. This is because WSNs have huge keys and computational requirements. ECC addresses these issues with strong encryption and smaller key sizes. Computing overhead and power utilization drop significantly [12]. With smaller key sizes, 256-bit ECC keys provide security equivalent to 3,072-bit RSA keys [13]. This smaller key lets resource-constrained WSN sensor nodes compute faster, utilize less memory, and save energy. ECC-based encryption consumes less energy since it transmits with less bandwidth. Due to its smaller key sizes, ECC simplifies cryptographic key management for decentralized, ever-changing wireless sensor networks (WSNs). Elliptic Curve Cryptography works with secure key exchange protocols and digital signature techniques such as EC Digital Signature Algorithm and EC Diffie-Hellman [14]. These protocols enable sensor nodes to exchange data, securely protecting its validity and integrity. Wireless sensor networks (WSNs) may achieve strict security requirements without impacting speed or power because ECC can conduct these cryptographic operations. ECC is the best cryptographic solution for wireless sensor networks (WSNs) because it allows trustworthy and secure sensor network operations in critical applications while considering processing power and energy limits [15].

Despite advancements in IoT security, authentication and intrusion detection systems in resource-constrained environments like IoT-based WSNs and IoMT networks struggle to reconcile computational efficiency, scalability, and full privacy protection. Traditional centralized solutions expose data and aren’t adaptive in real-time distributed environments, whereas PKI and password-based systems are computationally expensive and attackable. Current frameworks fail to integrate intelligent learning with lightweight security processes for adaptive detection, secure key management, and real-time reaction. The ECC-TLAF framework bridges the gap between safe, efficient, and intelligent IoT authentication and communication with lightweight ECC-based encryption and triple-layer authentication. It addresses these problems with scalable, privacy-preserving, low-latency security.

### 1.1 Motivation and novelty of ECC-TLAF

The rationale and motivation for using ECC in IoT-assisted WSNs is its distinctive capacity to provide robust security while utilizing minimal resources, rendering it particularly appropriate for resource-limited IoT devices. WSNs serve as the backbone of the IoT, enabling real-time data collection and transmission in diverse applications, including smart cities, healthcare, industrial automation, and environmental monitoring. However, WSNs are highly vulnerable to security threats due to their resource constraints, susceptibility to attacks, latency and energy overheads, and lack of multifactor authentication**. T**his research proposes the ECC-TLAF, which enhances security while optimizing energy efficiency and mitigates latency to address the abovementioned challenges.

Existing authentication and security methods in Wireless Sensor Networks (WSNs), notably those using RSA and standard PKI procedures, limit IoT application owing to practical constraints. Due to its enormous key sizes (1024–2048 bits), RSA-based encryption is computationally intensive and increases processor, memory, and energy consumption, despite its high security. This is problematic for WSN nodes with low processing and power. Due to certificate management complexity and key exchange frequency, public key infrastructure (PKI) systems increase communication latency and denial-of-service assaults. WSNs enabled by the Internet of Things have shifting topologies and mobility patterns that standard models can’t accommodate. Since next-generation WSN applications cannot use them for secure, lightweight, real-time communication, frameworks like ECC-TLAF are needed to provide high security with low resource utilization.

A novel approach to strengthening the security of WSNs is presented in this research, using ECC-TLAF, which combines biometrics, smart cards, and passwords. This innovative method achieves optimal resource utilization, high security, and energy efficiency by utilizing ECC’s lightweight multifactor authentication cryptography. By introducing dynamic key rotation and secure storage, the framework presents an automated key rotation mechanism and hardware-based trusted platform modules to mitigate key compromise risks in large-scale IoT deployments. To address essential vulnerabilities in IoT contexts, ECC-TLAF enhances attack detection and reduces processing time and latency compared to existing methods.

### 1.2 Contribution of the paper

The key contributions of the paper are:

a. An ECC-TLAF that integrates biometrics, smart cards, and passwords is to be developed to enhance WSN security.
b. ECC is to be implemented for secure key generation and encryption, with energy efficiency and computational overhead optimized.
c. A three-stage lightweight authentication mechanism is to be employed to improve attack detection and prevent unauthorized access.
d. ECC-TLAF is to be compared with traditional frameworks, with improved energy efficiency, lower latency, reduced computational complexity in storage, communication cost, and enhanced security being showcased.

### 1.3 Organization of the paper

The subsequent sections of the paper are organized, and Section 2 clarifies the existing research papers. The proposed approach of ECC-TLAF is concisely defined in Section 3 of this paper. The results and performance metrics of the recommended model are defined in Section 4. Section 5 summarizes the complete research article.

## 2. Related works

### 2.1 Artificial intelligence and intrusion detection for IoT-WSNs

Prasanth Aruchamy et al. [16] suggested the Artificial Intelligence (AI)-based Energy-aware Intrusion Detection System (IDS) and Secure Routing model for secured IoT-enabled WSNs. The suggested model runs the IDS to determine the different attack types. The next step in determining the need for protection is to include a decision mechanism based on game strategy in the suggested intrusion detection model. Finally, an energy-aware ad hoc on-demand distance vectors technique has developed a secure routing solution across several nodes. When tested against existing intrusion detection algorithms, the suggested model outperforms them with a 95% detection accuracy and a 38% decrease in energy use.

### 2.2 Cryptographic solutions and mutual authentication schemes

Alaa Allakany et al. [17] recommended the Mutual Authentication Scheme (MAS) for Enhancing Security in ZigBee WSN. The suggested method strengthens ZigBee’s cryptography by enhancing the encryption procedure of a standard AES without requiring asymmetric cryptography. The author uses bitwise exclusive OR operations to strengthen cryptography and a secure one-way hash function operation during device-to-device (D2D) and device-to-trust centre (D2TC) mutual authentication. After the parties using ZigBee have completed authentication, they may communicate a secure value and agree upon a shared session key. This encrypted value and device data are then utilized for conventional AES encryption. This technology strongly prevents cryptoanalysis attacks on encrypted data. The author showed how the suggested system maintains efficiency compared to eight competing systems. This research evaluates the scheme’s security, communication, and computation costs. Ravi Kumar et al. [18] proposed safe user authentication strategies for IoT-enabled WSN data transfer. The recommended technique reduces processing costs using biological data, hash algorithms, and XOR operations. A secure session key, biometric data, and password updating ensure forward and backward confidential discussion. The real-or-random (ROR) paradigm evaluates the scheme and session key’s security. With the AVISPA simulation tools, the author verifies the user authentication method. Experiments show that the recommended solution outperforms multifactor, multi-gateway authentication schemes.

### 2.3 Blockchain and asymmetric cryptography in secure IoT networks

Hosny H et al. [19] explored the IoT Network Security Framework using Blockchain. This article uses blockchain technology and the LEACH algorithm to secure IoT networks. LEACH pregroups IoT devices, and a cluster head aggregates and forwards data. The approach integrates blockchain technology’s cryptography and basic notions to improve security. Layers and clusters are created via a blockchain simulator module and LEACH clustering-based routing protocols. The LEACH algorithm boosts power efficiency, simplifies cluster data management, and ensures transaction immutability, transparency, and integrity. This model may be tested and tweaked using a simulator to evaluate security-improved IoT network models. The updated LEACH technique outperforms prior methods when the final node failed after 1868 transactions. The suggested architecture achieves a throughput of 2.75 and a state rate of 0.058%.

Bhanu Priyanka Valluri and Nitin Sharma [20] introduced Exceptional Key-based Node Validations for Secure Data Transmission utilizing Asymmetric Cryptography in WSN. This study achieves secure data transmission from source to destination by validating nodes, encrypting data, and decrypting data in the WSN. This study proposes a method to ensure sensor data security during transmission and after nodes receive it. For many WSN uses, public key cryptography is the best and most reliable option because it incorporates key administration, a more solid and productive form of key generation and trading handling. The suggested paradigm has a node validation accuracy of 98% and an encryption and decryption accuracy of 98.6%.

### 2.4 Multifactor authentication and routing algorithms

Albandari Alsumayt et al. [21] deliberated the four-factor authentication scheme for Effective security levels in WSNs over the IoT. In this approach, fingerprint, random number (nonce), timestamp, and key all undergo homomorphic encryption. The implementation results confirm the usefulness of the suggested technique in authenticating users in systems. Banking, healthcare, education, internet shopping, and many more fields and organizations may benefit from the suggested approach. The author assumed that federated learning decision-makers would evaluate the readings and determine the authentication success or failure. Simulation findings demonstrate the efficacy of the suggested approach, underscoring the significance of using this protocol in authentication domains.

Vanita Verma and Vijay Kumar Jha [22] suggested the Mutation-grounded Multi-Layer Perceptrons (MUMLP) for IoT-enabled WSN with the Assistance of Cluster-Based Secure Optimal Routing. The best Cluster Heads (CHs) are chosen for Data Aggregations (DA) using the Boltzmann Selection Probability-centric Gravitational Search Algorithms (BSP-GSA). Following the network’s node startup, several tasks are carried out. The next step is to join a neighbouring CH with the non-cluster members so they may create a cluster. Data collected from non-cluster members is encrypted using the Improved ECC technique and then protected by the CH. The encrypted information is sent to the base station via the best path determined by a Deep Learning (DL) that considers a new fitness function. A cloud server stores the data in the BS for user access. Blockchain (BC) authentication is used to avoid unwanted access. This authentication ensures that only authorized users may access the data. Simulations are performed to assess how effective the suggested method is. When compared to the current techniques, the outcomes demonstrate that the given algorithm transmits data safely and with consideration for energy.

### 2.5 Three-factor authentication and unequal clustering in IoT-WSNs

Shreeya Swagatika Sahoo et al. [23] presented the three-factor-based data transmission authentication schemes (TDTAS) for 5G WSN for IoT System. The Real-or-Random (RoR) paradigm shows the formal security analysis. According to the informal security study, the system accomplishes additional security features and is secure against numerous known attacks. According to the security research findings, TDTAS resists the most known security threats and vulnerabilities. In contrast to previous methods, the suggested one has its formal security analysis validated utilizing the RoR model. A casual security study shows that the system is strong and secure. The author has used a well-recognized AVISPA tool to verify our approach formally. There is a considerable decrease in the transmission and storage expenses associated with the suggested strategy compared to similar schemes. Indresh Kumar Gupta et al. [24] investigated the Fire Hawk Optimization-based Unequal Clustering Scheme for Hotspot Mitigations (FHOUCS-HSM) in IoT-enabled WSNs. The FHOUCS-HSM method is based on the foraging behaviours of brown falcons, whistling kites, and black kites. This study is innovative because the FHO method is designed to handle uneven clustering. Furthermore, a fitness function is computed using the FHOUCS-HSM model for cluster heads (CH) selection and unequal cluster size. By comparing it to other models, the comparative study shows that the FHOUCS-HSM method is superior.

### 2.6 Energy-efficient routing and secure data communication

Divya Gupta et al. [25] proposed Energy Effective Data Communication Schemes (EEDC) using Region-based Hierarchical Clustering for Effective Routing (RHCER) for secure and energy-aware routing decisions in IoT-based WSNs. The sensors used to gather data for IoT applications collect crucial information and use a multi-criteria decision function to choose cluster heads. The author compared EEDC’s performance to other energy-efficient protocols already used for several metrics. Using the proposed technique, the simulation results demonstrate a 31% decrease in energy consumption in sensor nodes and a 38% improvement in packet loss ratio.

Sohail Saif et al. [26] suggested the Dynamic Lightweight Symmetric Encryption Algorithm (DLSEA) for secure data transmission in IoT-based pervasive sensing applications. DLSEA is implemented on the ATmega2560 and ATmega328P microcontrollers using the Proteus simulation tool, which encrypts data using a 64-bit block cipher and a 64-bit temporal secret key. Many tests were carried out with payloads of varied sizes to measure the avalanche effect, execution time, energy consumption, RAM use, and ROM usage. According to the results, DLSEA is 47% quicker than PRESENT, another comparable technique, on the ATmega2560 for data sizes ranging from 128 to 512 bytes, taking 2.49 to 3.27 ms. With a 41.81% drop in RAM utilization and a 57.19% drop in ROM usage compared to PRESENT, DLSEA is 46% more energy efficient. The findings of the experiments support the idea that DLSEA passes all three tests for sensitivity: the plaintext test, the key test, and the strict avalanche criterion test (i.e., around 50%).

Sohail Saif et al. [27] recommended the Timestamp-based Secret Key Generation (T-SKG) scheme for a secure data transmission framework for IoT-enabled healthcare. Results from MATLAB and Java simulations show that the T-SKG scheme is robust against brute force, guessing, and birthday assaults. When the attacker knows the key sequence pattern, a guessing attack has a 9% probability of compromising the key, but the scheme is protected against brute force and birthday assaults during a certain timescale. When the MySignals sensor kit is used with a healthcare framework, the T-SKG scheme ensures the safe transmission of crucial health data. The Data Encryption Standard (DES) is used for secrecy, and various Cipher Block modes (ECB, CBC, and CTR) are included.

In Table 1 the representation of symbols indicates ✔✔✔-highly effective, ✔✔-moderately effective, ✔-effective, ➖ Neutral/ moderate, ✖-limited/high overhead. While blockchain and AI-based solutions provide strong security [16,19], they may affect IoT networks with limited resources due to their higher computation overhead. In comparison, lightweight encryption methods [27,28] guarantee security with minimal computation cost and are tailored for low-power IoT devices. Due to the additional authentication processes, multifactor authentication systems [17,18,21,23] offer enhanced security resilience against assaults, although they may cause slight computational overhead. Secure data transmission and energy efficiency are the main goals of optimization-based clustering techniques [24,25], which also aim to increase the lifetime of networks. Considering their unique requirements and limitations, these findings are useful for choosing the best security framework for IoT-WSN environments.

**Table 1:** Comparison of Security & Optimization Techniques.

Reference	Approach	Efficiency	Security Strength	Energy Consumption	Computation Overhead	Scalability	Unique Contribution
[16]	AI-Based IDS & Secure Routing	✔✔	✔✔✔	✔✔	✖	✔✔	AI-driven adaptive intrusion detection
[17]	Mutual Authentication (MAS)	✔✔	✔✔✔	✔	➖	✔	Enhanced AES-based ZigBee authentication
[18]	Biometric-Based Authentication	✔✔	✔✔✔	✔	✖	✔✔	Secure session key with biometrics
[19]	Blockchain + LEACH	✔	✔✔✔	✔✔	✖	✔✔✔	Hybrid blockchain-secured clustering
[20]	Key-Based Node Validation	✔✔	✔✔✔	✔	➖	✔	Public key cryptography-based security
[21]	Four-Factor Authentication	✔✔	✔✔✔	➖	✖	✔	Homomorphic encryption for authentication
[22]	Mutation-Based MLP Routing	✔✔	✔✔	✖	✔	✔✔	AI-driven secure routing optimization
[23]	Three-Factor Authentication	✔✔	✔✔✔	✔	✔	✔	AVISPA-validated low-cost authentication
[24]	Fire Hawk Optimization (FHOUCS-HSM)	✔✔	✔✔	✔	➖	✔✔✔	Nature-inspired clustering optimization
[25]	Region-Based Hierarchical Clustering	✔✔	✔✔	✔✔	✖	✔✔	Secure routing with adaptive CH selection
[27]	DLSEA Lightweight Encryption	✔✔✔	✔✔	✔✔✔	➖	✔✔	Optimized for low-power microcontrollers
[28]	Timestamp-Based Secret Key (T-SKG)	✔✔	✔✔✔	✔	➖	✔✔	Resistant to brute-force attacks

An intrusion detection system for Internet of Things (IoT)-based WSNs is suggested by Anwar et al. [28], which combines a federated learning (FL) architecture with long short-term memory (LSTM) networks. To improve detection accuracy and decrease false positive rates, the model enables distributed training without sharing raw data, thereby preserving anonymity. With impressive results on WSN-DS, CIC-IDS-2017, and UNSW-NB15 datasets, the FL-LSTM method shows great promise for scalable and privacy-preserving intrusion detection in IoT settings, surpassing typical centralized models in accuracy, F1 score, and RMSE.

Asif et al. [29] proposes a two-stage dual authentication architecture for safe sensor-to-server communication in IoMT scenarios to avoid the limitations of passwords or PKI. ECDH for key exchange during registration and AES-GCM for safe real-time data encryption make the framework suitable for sensitive data. The simulation findings show strong resilience to man-in-the-middle, replay, and brute-force attacks and decreased encryption/decryption time (over 45%), computational cost (45.38%), and latency (28.42%) compared to earlier techniques. The design ensures medical IoT device packet delivery security and performance.

Ullah et al. [30] introduces the DeepTrust framework on which a deep learning-based trust and reputation management system for IoT environments, aimed at enhancing security by accurately distinguishing between trustworthy and untrustworthy devices. Experimental results demonstrate significant performance improvements over traditional methods, with gains of 15% in accuracy, 20% in precision, and 18% in recall. Designed for real-time adaptive assessments, DeepTrust addresses limitations in conventional models, particularly in dynamic and large-scale IoT networks. Future enhancements may include reducing computational complexity, supporting incremental learning, improving data privacy, and expanding applicability across diverse IoT scenarios, making it a strong candidate for universal trust management in IoT. Table 2 provides the survey comparison

**Table 2:** Summary of Recent Authentication Protocols in IoT Environments.

Method	Core Technology	Application Domain	Key Features	Relevance to ECC-TLAF
CMAF-IIoT [31]	Chaotic Map & ECC	Industrial IoT	Lightweight chaotic mapping, ECC key exchange	Shows ECC viability in industrial scenarios
REAP-IIoT [32]	Elliptic Curve Cryptography	IIoT	Resource-efficient, two-factor authentication	Reinforces ECC’s efficiency and low overhead
Lightweight Protocol for Medical IoT [33]	ECC + Access Control	Connected Healthcare	Privacy-preserving access, low-latency authentication	Highlights healthcare-specific lightweight needs
PUF-Based Auth. for Smart Healthcare [34]	Physical Unclonable Functions	Smart Healthcare	Hardware-level authentication, energy-efficient	Emphasizes hardware-aided trust enhancement
RAAF-MEC [35]	Anonymous Authentication	MEC-IoT	Anonymity, reliability, MEC integration	Aligns with ECC-TLAF’s privacy and mobility goals
UAV-IoAV Auth Protocol [36]	ECC + Lightweight Cryptography	UAV-assisted IoAV	Privacy-preserving, lightweight authentication	Demonstrates ECC’s adaptability in aerial IoT with constrained environments
Autonomous Vehicular Auth [37]	ECC + Privacy-Preserving Protocols	Autonomous Vehicles (IoT)	Provable security, privacy preservation, low-resource overhead	Validates ECC use in vehicular systems requiring real-time secure access
Trust-Blockchain Info Sharing [38]	Blockchain + Trust Score + DPoS	Internet of Vehicles (IoV)	Trust-based miner selection, secure information sharing	Supports ECC-TLAF goals via trust-integrated blockchain in vehicular systems
BTC2PA [39]	Blockchain + ECC + Conditional Authentication	Connected Vehicles (VANET)	PKI with digital signatures, trust score with reward-punishment model	Strengthens ECC-TLAF with scalable, privacy-preserving vehicular authentication

The proposed ECC-TLAF framework is tested against many IoT and WSN baseline authentication methods. RSA-based authentication schemes have high computational overhead but strong security; ECDSA-based lightweight authentication models have better efficiency but may not have advanced attack resistance; and traditional PKI-based models have complex certificate management and aren’t good for resource-constrained IoT environments. We compare ECC-TLAF to recent frameworks like PUF-based protocols and dual-authentication models to highlight its energy economy, decreased latency, attack resilience, and end-to-end data security. The current IoT authentication literature uses these baselines, and ECC-TLAF balances performance with good multi-layer security.

There are several security risks that current methods in IoT-assisted WSNs are susceptible to. These include man-in-the-middle attacks, replay attacks, and side-channel attacks. The security and privacy of sensitive information are jeopardized in man-in-the-middle attacks because the attackers can intercept communication lines. To get unauthorized access, replay attacks capture and reuse legitimate authentication messages, which exploits authentication failures. To get sensitive information, side-channel attacks aim against the physical implementation of cryptographic methods, such as ECC. To counter these risks, the suggested solution employs a triple-layer authentication technique, which makes it much more difficult for intruders to sneak into the system undetected by checking the identities of devices, networks, and servers on numerous levels. A lightweight symmetric encryption protocol with ECC for secure key exchange makes it an effective data encryptor that reduces vulnerability to replay and side-channel attacks. This tiered method ensures data integrity and privacy throughout the network, strengthening authentication and encryption while enhancing resistance against typical IoT security threats.

Based on the investigation, there are several challenges with existing models in achieving a high energy efficiency ratio, security enhancement, attack detection and decreased processing time and latency. Hence, this paper proposes Elliptic Curve Cryptography and Triple-Layer Authentication Framework (ECC-TLAF) to improve the security performance of WSNs effectively.

## 3. Elliptic curve cryptography and triple-layer authentication framework (ECC-TLAF)

In recent years, communication between people and smart components or Internet-based components has been made possible by the IoT, an advanced applied science. Additionally, IoT enables the integration of real-world and digital components, which various software, hardware, and interface technologies may manage. Many Internet of Things (IoT) approaches improve our daily lives in many ways, yet they have serious vulnerabilities. Conventional approaches are open to a wide variety of threats. Therefore, it is still very difficult to develop reliable security solutions for the IoT, and the biggest concern with this security measure is how to transmit sensitive data safely. Due to the specific components of the IoT, such as WSN nodes and RFID tags, it is not possible to use such generic alternatives because of the structure’s diversity. Experts aimed to overcome these constraints by creating lightweight protocols for the IoT paradigm. One of the most talked-about and active areas of study in this field is the authentication and session key agreement protocol. There are numerous benefits to using IoT in everyday life, yet there are significant privacy risks. Securely transmitting sensitive data via the network poses the greatest threat to the IoT. Security for the IoT primarily relies on two technologies: device authentication and secret key storage. Current key generation and authentication methods rely heavily on traditional cryptography, which comes with the drawbacks of expensive secret key storage and advanced cryptographic algorithms. Most methods rely on a one-factor authentication strategy, which might pose some privacy risks. Several academics have recently sought to implement an asymmetric encryption-based secure authentication mechanism. Perception, conveyance, and application are the three pillars of an IoT system. IoT components provide a lightweight mutual authentication protocol that is ECC-enabled to protect the system against IoT vulnerabilities while keeping communication overhead to a minimum. Hence, this paper proposes ECC-TLAF to improve the security performance of WSNs effectively.

**Fig 1:**
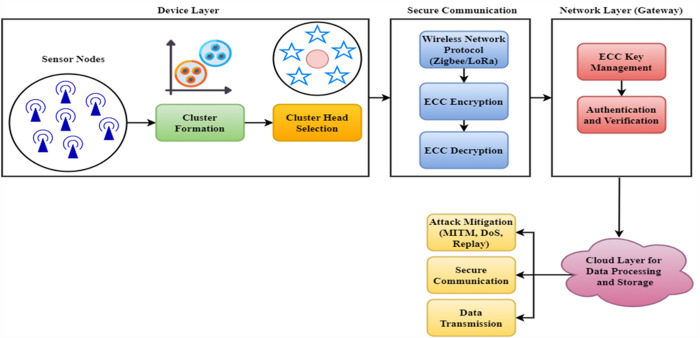
Proposed ECC-TLAF Model.

Fig 1 shows the proposed ECC-TLAF model. A sensor node or Internet of Things device may collect and send data about the environment to a network. Efficient key generation, encryption, and data decryption are made possible by the ECC module, which is included in every device. Because of their limited resources, IoT devices benefit greatly from ECC’s device-level cryptographic operations, guaranteeing lightweight cryptography. Each device creates its own set of ECC private and public keys for secure authentication and communication. The Secure Communication Layer employs ECC-Based communication to keep information private and undeleted, primarily concerned with data encryption. IoT nodes encrypt to ensure sensitive data is secure while being sent to the network gateway. ECC-based encryption is very advantageous for low-power Internet of Things scenarios because of its decreased key sizes and high level of security. Since the network gateway encrypts data with its private key, only authorized and certified devices may transmit data. Amid the network layer, the Gateway connects IoT devices to the cloud. This gateway must handle ECC key exchanges and ensure data packet integrity. Security procedures include packet verification, ensuring that any Internet of Things devices linked to it are genuine. Network traffic between the gateway and Internet of Things nodes is encrypted to prevent eavesdropping and MITM attacks. The design requires Triple-Layer Authentication. Approval of devices, networks, and apps before data access improves security. The gateway requires ECC-based digital signatures for self-authentication on all IoT devices. This technique checks the device’s ID before the network connection. TLS and OAuth2.0 provide network-level security after device authentication. This protects session administration and network access from unauthorized devices. When a user or application requests IoT data, the final layer verifies their identity. Role-based access control (RBAC) prohibits illegal data access or changes even with successful lower-level authentications. Data is processed, stored, and aggregated in the cloud after network transmission. The data is encrypted using ECC in the cloud to prevent unauthorized access to sensitive information in transit and storage. This information is accessible only to authorized users or apps that have completed the three-factor authentication procedure. The design prevents attacks like MITM, Denial of Service (DoS), and Replay. Secure and robust communication against typical IoT-related threats is achieved via the combined efforts of ECC and the triple-layer authentication mechanism.

To ensure the network remains intact, if one gateway is compromised, another may easily take over its function thanks to the failover measures in place. This helps to reduce the impact of a single point of failure at the gateway. This study provides multi-layered authentication at the gateway level, making it very difficult to get illegal access even if the gateway is targeted by requiring verification from many sources for each access. To further reduce data exposure in the case of a breach, the gateway does very little data processing and does not save any sensitive information locally. Its primary task is to act as a pass-through entity for encrypted data. On top of that, end-to-end encryption is used for all vital data flowing through the gateway. This ensures the data is secure regardless of the gateway’s security status.

The IoT device first generates an ECC key pair consisting of a private key stored on the device and a public key shared with the Network Gateway. Mutual authentication is made possible via the public key exchange. The Network Gateway then passes the device’s public key onto the Authentication System, which uses ECC-based signature validation to confirm the device’s validity. Once verified, the Network Gateway and Authentication System take on a mutual authentication operation to provide a secure communication route. Following successful authentication, the IoT device encrypts the data using its Elliptic Curve Integrated Encryption Scheme (ECIES) private key and employs an ECC-based encryption method. Due to this procedure, only entities with the matching ECC public key can now decipher the data in the ciphertext. The encrypted data is sent to the cloud server via the network gateway. Additional security measures, such as hash-based integrity inspections, are implemented at the Cloud Server to avoid misuse. Anyone authorized with the ECC public key may decode the encrypted data and access the stored information. Using the ECC public-private key connection, this decryption procedure guarantees that authorized individuals may only access data. Encryption and decryption using ECC improves data confidentiality and integrity during storage and transmission while reducing computational costs. ECC uses smaller keys to provide a similar level of security as non-ECC (like RSA). Therefore, among public-key cryptosystems, it may be a suitable target for WSN environments. Many devices in limited contexts, such as the IoT and WSN, rely on ECC. ECC is useful because it achieves the same encryption difficulty as other asymmetric cryptography systems, using much smaller keys and requiring much less processing. For example, the encryption difficulty of the RSA method with 1024-bit keys is similar to that of the ECC algorithms with 160-bit keys. This discernible variation in key size decreases encryption’s storage and computing requirements. Consequently, ECC boosts the capabilities of low-computation devices, making them more effective. Key exchange mechanisms and methods for securing communications between senders and receivers are two aspects that distinguish ECC implementations. To top it all off, ECC prevents forgeries by signing plain texts, which keeps messages unaffected. The elliptic curve group operation is signified as "+" for adding 2 point.

Given two distinct points on the curve let, $Q\;=\;(y_{1},\;x_{1}and\;P\;=\;(y_{2},\;x_{2})$. Thus, the adding of $Q+P=\;(y_{3},\;x_{3})$ can be articulated as $\left(y_{1},\;x_{1}+\;(y_{2},\;x_{2})\;=\;(y_{3},\;x_{3}\right)$, where $x_{3}$ and $y_{3}$ are derived based on elliptic curve addition rules. In another case, where Q = P, the cluster operations are signified as multiplications β × Q on elliptic curves ended ${\mathbb{Z}}_{q},\;q\;>\;3$ that fulfil Expression (1), such that $b,a\;\in \;{\mathbb{Z}}_{q}$ and $4\;\times \;b^{3}\;+\;27\;\times \;a^{2}=\;0\;mod\;q$ where β is integer values. For example, applying point doubling can be given as,


$$Q+\;Q\;=\;(y_{1},\;x_{1})\;+\;(y_{1},\;x_{1})\;=\;2Q),\;\;3Q=(y_{1},\;x_{1})\;+\;\;(y_{1},\;x_{1})\;+\;\;(y_{1},\;x_{1})$$

likewise, 3Q is equivalent to $\;(y_{1},\;x_{1}+\;\;(y_{1},\;x_{1})\;+\;\;(y_{1},\;x_{1})$ essential for efficient key generation in cryptographic systems. The ECC should be a non-singular curve (i.e., no numerous roots).


$$\;\begin{array}{c} y^{2}\equiv x^{3}+ax+b(mod\;q);where\;a,b\in {𝔽}_{q}\\ ax^{2}+y^{2}=1+dx^{2}y^{2}\\ E:y^{2}=x^{3}+ax+b,\;where\;4a^{3}+27b^{2}\neq 0 \end{array}\;\;\;\}$$ (1)

Key operations in EC are cluster multiplication operations. For dQ, this denotes to d periods of the adding of points Q, which outcomes in novel points $\left(y_{i}\;,\;x_{i}\right)$. The private keys are maximum integers d, and values of the multiplication operations $\left(y_{i}\;,\;x_{i}\right)$ is recognized as public keys. Being aware of the public key point $(y_{i}\;,\;x_{i}\backslash )$makes ECC a difficult mathematical challenge, and starting points Q, it is impossible to calculate d in a polynomial period.

The nine-step process outlined in the proposal begins with setting up the necessary system parameters. It continues with encoding the plaintext message, finding the point on the EC that map to the message, encrypting those points, passing the accumulated mapped point as cipher texts, and lastly, doing the contrary of the prior steps by checking and decoding the expected cipher texts, deciphering the point, and finally, producing plaintext from encrypted points. The proposed ECC encryption system is the primary result of this research, as it is both efficient and secure. It makes it easier for parties to communicate securely by generating a shared key that they can all use to encrypt the communications they send to each other. The first step is to create a shared key, and many new studies have ignored the significance of this step in encrypting messages sent between participants. An improved secure way to encrypt plaintext and map it to EC is shown in this paper. Because the ECC is an essential component of any system for protecting group communication, our research zeroes in on its three initial stages (e.g., IoT environments). This step provides the variables to secure communication amongst all participants by establishing private and public keys. The shared group key $hk_{s}$ is partially created at this stage and is utilized to encode the message shared amongst cluster members. The edge needs to develop and preserve the shared cluster keys $k_{s}$. This allows the persons to decode cipher texts efficiently. Thus, the edge generates the first $k_{s}\;$utilizing its $G_{1}\left({id}_{ed}\right)$ and private random keys. Similarly, Equation (2) updates $k_{s}\;$ for each new node that enters the group.


$$k_{s}=G_{1}\left({id}_{mj}\|N_{m}\right)\;⨁\;k_{s}$$ (2)

To prevent key reuse attacks and enhance randomness, a nonce $\|N_{m}\;$ generated at each session denoting with concatenation, ensuring uniqueness in each session. Ensuring that newly joined nodes cannot decipher the encrypted text received before they joined the group using $k_{s}$ is crucial for maintaining forward and backward secrecy. The decryption of ciphertext delivered after a node leaves the group is impossible. The suggested system does this by empowering each node to carry out certain activities that keep $k_{s}\;$constant. For new nodes to join groups, the edge directs the joined nodes’ hash ID $G_{1}({id}_{mj}\backslash )$ to every present node.

The suggested approach necessitates the generation of three distinct types of keys. Data decryption begins with the generation of a public key. Next, it’s necessary to create a private key to decode the data. The first step in encrypting data is to create the public key. Afterwards, a private key is created to decode the data. Finally, the public and private keys combine the elliptic curve points to form the secret key. Considering points $A_{s}$ to be base points on the curve. Select random numbers between 0 and m−1 to create private keys $Pr_{k}$. The public keys ${Pu}_{k}$ is created as demonstrated in Equation (3) and (4):


$${Pu}_{k}=Pr_{k}*A_{s};{Pu}_{k}=\prod \left(Pr_{k},\;A_{s}\right)$$ (3)

The secret key $W_{k}$ is produced by summing ${Pu}_{k}$ public key, $Pr_{k}$ private key, and $A_{s}$ as system generated elliptic curve parameter, $\begin{array}{c} \prod \left(Pr_{k},\;A_{s}\right) \end{array}$ denotes the multiplication accumulation across all values of ${\mathrm{Pr}}_{k}$ and $A_{s}$ which is transcribed as Equation (5):


$$W_{k}=\sum \left({Pu}_{k},Pr_{k},A_{s}\right)$$ (5)

After the key is generated, the values determined from the IoT devices are encoded. The encoded data comprises two ciphertexts statistically inscribed as equations (6) and (7).


$$D_{1}=\left(W_{1}*A_{s}\right)+W_{k};where\;W_{1}\;as\;1\leq W_{1}\leq m-1$$ (6)

$$D_{2}=N+\left(W_{1}*{Pu}_{k}\right)+W_{k}$$ (7)

As shown in equations (6) and (7) where $D_{1}\;$and $D_{2}\;$are ciphertexts 1 and ciphertexts 2; $W_{1}\;$is random numbers between 1 and m−1; and N specifies the message, $A_{s}$ as the elliptic curve system parameter, and $W_{k}$ as the secret key. The actual data can be determined from the decryption process with $\left(\frac{D_{2}-Pr_{k}}{D_{1}}\right)$ underogoes the encryption operation, $W_{k}$ is subtracted to extract the original message.


$$N=\left(\frac{D_{2}-Pr_{k}}{D_{1}}\right)-W_{k}$$ (8)

**Fig 2:**
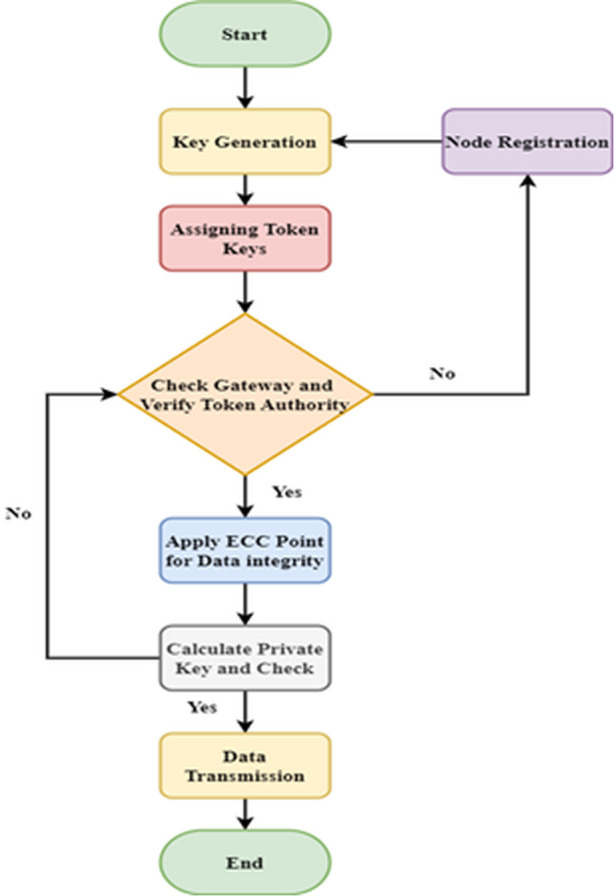
Token-based security using ECC.

Fig 2 shows the token-based security using ECC. Private keys are generated using an elliptic curves point algorithm for each node. From the generated key values, the preceding 8-byte succession is extracted. Performing the reverse series operation in extracted 8-byte series. Reversed series succession is encrypted using a private key to protect data integrity. The generated private and public keys are applied to the commutative property, and the commutative function states are in the Equation. The source nodes send the public key, the destination node replies with private keys, and gateways verify the key. If the key matches, the nodes can be labelled legitimate, and the data transmission is done with integrity. If the generated key does not match, the nodes are proved illegitimate, and the data transmission ends. This study has implemented several layers of protection to safeguard against attacks that target the underlying network infrastructure, as we have identified its potential weakness. Data stays encrypted from the IoT device to the cloud server regardless of network circumstances since end-to-end encryption is enforced. This method lessens the effect of security holes at the network level. In addition, our network infrastructure is equipped with intrusion detection systems (IDS) and multi-layered authentication to identify and address any irregularities instantly, decreasing the probability of successful assaults. By dividing the network into zones and equipping each zone with regulated access points, this study can ensure that prospective attackers cannot move around the network in case of a breach. These safeguards work together to keep data and IoT devices safe, even if the network is susceptible to attack.

An optimum cluster head selection function based on many criteria forms the basis of the first level. The next step is constructing various clusters using the nodes with the lowest energy consumption. Level two of the suggested energy link-effective routing scheme involves making the routing channels more stable over an extended period, which stops the wireless connection from behaving up from the CHs to the BS. The suggested model uses the node energy $e_{j}$, Signal-to-Noise Ratio ${SNR}_{j,BS}$, and distances to BS $d_{i,BS}$ as multi-criteria decision-making functions f(m) for selecting CHs, as provided in equation 9. The suggested framework aims to use SNR to determine the signal intensity and effectively enhance delivery performance. Signal-to-noise ratio, or SNR, measures how well a signal can be detected relative to ambient noise. Let us deliberate on that ${RSSI}_{j}\;$is the received signal power indicators and ${Bm}_{j}\;$signifies the noise for the connection j consequently, the values of ${SNR}_{j}$ can be calculated by ${RSSI}_{j}/{Bm}_{j}$. The connection with the least ${SNR}_{j}\;$values are selected as more suitable for data transmissions.


$$f(m)=\alpha \cdot e_{j}^{\eta_{1}}+\beta \cdot \left(\frac{1}{d_{i,BS}}\right)+\lambda \cdot \left(1+\frac{1}{{SNR}_{j}^{\delta }}\right)+\mu \cdot \log\left(1+e_{j}^{\eta_{2}}\right)+\nu \cdot e^{-\theta d_{i,BS}}$$ (9)

The value of f(m) is standardized in the range of [0 . . . 1] utilizing 1−f(m). $\log\left(1+e_{j}^{\eta_{2}}\right)$ ensures a smooth scaling effect for residual energy, and $e^{-\theta d_{i,BS}}$ penalizes long-distance transmissions exponentially, reducing energy wastage. The α,β,λ,μ,ν,θ are tunable parameters weight parameters allows for adaptive tuning based on real-time network conditions, and $\eta_{1},\eta_{2},\delta$ introduces higher-order nonlinearity.

BS maintains a global database containing WSN sensor data in the suggested model. The data stored include the locations of nodes, their distances from BS, residual energies, and SNR aspects. After examining the stored data, BS calculates a normalized score by evaluating f(m). Then, as a starting point for the cluster, BS selects the sensor node with the greatest local score. So, using f(m), BS assigns each entry to a node that will serve as the cluster head. Every other node not designated as a cluster head is just another regular node. After that, BS adds the one-hop node from every CH to its membership and changes the global database appropriately. Under the suggested architecture, each WSN sensor communicated with its cluster head to collect the necessary data. Users can access data about farmland for further analysis when CHs utilize a single-hop technique to send the information to BSs, which connects to the Internet. Algorithm 1 shows the pseudocode for TLA with ECC encryption.

**Algorithm 1: Pseudocode for TLA with ECC Encryption and Decryption**

Define the elliptic curve over a finite field

Choose a large prime number p and curve parameters a and b

Select a base point G on the curve with order n

function GenerateKeys():

d = Random(1, n-1)

Q = d * G

return (d, Q)

function AuthenticateAndEncrypt(M, Q_device, user_credentials):

// Layer 1: Device Authentication

if VerifyDevice(Q_device) == False:

return "Device Authentication Failed"

// Layer 2: User Authentication

if CheckUserCredentials(user_credentials) == False:

return "User Authentication Failed"

// Layer 3: Session Authentication & Encryption

k = Random(1, n-1)

C1 = k * G

C2 = M + k * Q_device

return (C1, C2)

function DecryptAndManageSession(C1, C2, d_device):

S = d_device * C1

M = C2 - S

InitiateSecureSession(M)

return M

**Fig 3:**
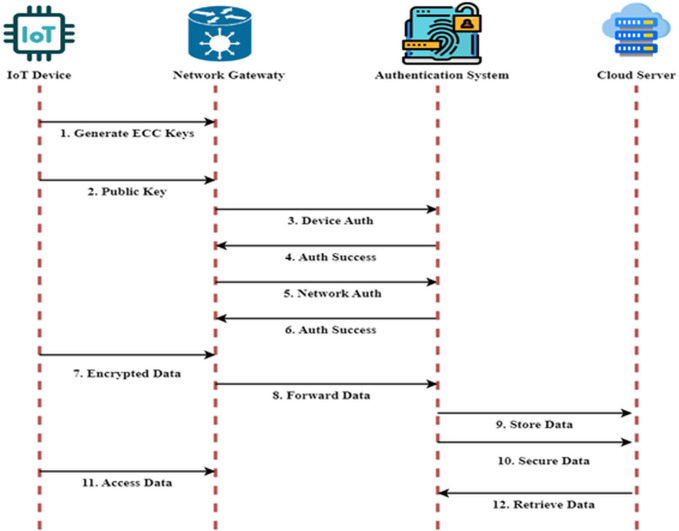
Sequence Diagram.

Fig 3 shows the sequence diagram. As a first step, the Internet of Things device must prepare to interact with the network’s gateway and generate its ECC key pair. The device’s public key is sent to the Network Gateway, which then passes to the Authentication System for validation. Digital signatures based on ECC are used to confirm the identification of the IoT device in the first protection. The network gateway establishes a secure connection using protocols such as TLS or OAuth2.0 to validate the device. Because of this additional layer, the device and the network can securely interact among themselves. Verifying the credentials of the person or program that gained access to the data is the third kind of authentication that RBAC often manages. After the authentication procedure has been finished, the Internet of Things device will first encrypt the data it has acquired by using the public key connected with the ECC of the gateway, and then it will transfer the encrypted data along its path.

The primary use of ECC is the generation of public-private key pairs, which are essential for implementing digital signatures and safe key exchange. Other cryptographic methods, like AES, can encrypt and decrypt data because ECC establishes shared keys, which provide safe communication channels. Because of this, ECC is often used in conjunction with symmetric encryption algorithms to secure data, with ECC handling the key exchange. To guarantee secure key management and data confidentiality without overwhelming the limited computing resources of IoT devices, this technique utilizes ECC’s efficiency in resource-constrained contexts.

After the data has been decrypted by the Network Gateway and checked to ensure it has not been changed, it is sent to the Cloud Server for information storage. Since all data is encrypted before, during, and after processing in the cloud, security is of the utmost importance. With the help of Tri-Layer Authentication, you can be certain that only authorized persons or applications can access the information. For less-than-ideal Internet of Things setups with limited resources, this architecture will secure Internet of Things devices, network transmissions, and cloud-based data storage against various cyber threats, including MITM attacks and replay attacks. The suggested solution employs Elliptic Curve Cryptography (ECC) for effective key generation, which allows us to tackle important management issues. ECC reduces the computational burden on IoT devices with limited resources by providing good security with reduced key lengths. A public-key infrastructure (PKI) architecture is used for safe key distribution.

Algorithm: 2 ECC-TLAF Secure Communication Against MITM Attacks

1: Generate $\left({k_{private},k}_{public}\right)=ECC\cdot KeyGen()$

2: Send $SN\to NG:K_{public}$

3: $A\;intercepts\;k_{public}$

4: generate $A\to NG:k_{A}^{public}$

5:$AS\;verifies:ECC:verify\left(k_{public},k_{A}^{signature}\right)$

6: if valid $k_{session}=k_{private}\times k_{A}^{public}$

7: else reject authentication

8: encrypt $C=ECC\cdot encrypt\left(k_{session},\;data\right)$

9: send SN→NG:C

10: attacker intercepts A→C

11: receives NG←C

12: decrypt $ECC\cdot decrypt(k_{session},C)$

13: NG successfully retrieves data

14: attack prevented

The proposed ECC-TLAF successfully defends against MITM by utilizing authentication procedures, session key agreements, and ECC using parameters such as ECC key pair $\left({k_{private},k}_{public}\right)$, public key SN, Attacker intercepts $k_{public}$ to generate and sends its own public key: A. By combining elliptic curve cryptography (ECC) with a three-layer authentication system, ECC-TLAF guarantees safe Communication in WSNs. Through the process of authenticating public keys and creating a secure session key, it safeguards the sensor node from man-in-the-middle assaults. An attacker could not decipher encrypted data during transmission without the session key. The framework improves security for IoT devices to make the most of limited computing resources.

The algorithm steps provide a strong security foundation against various cryptographic threats. It ensures resilience against MITM attacks by employing ECC for secure key exchange and authentication. The inclusion of signature verification (Step 5) prevents impersonation attacks, ensuring that only legitimate nodes can establish secure communication. Brute-force attacks are mitigated due to the large key space of ECC, making key guessing computationally infeasible. Replay attacks are countered using dynamically generated session keys (Step 6), ensuring that intercepted keys cannot be reused. Additionally, data confidentiality is preserved through encryption (Step 8), preventing eavesdropping. This scheme effectively withstands cryptographic threats by validating authentication and secure transmission, ensuring a robust and efficient security framework.

The recommended ECC-TLAF architecture prevents privilege insider attacks with multi-layered authentication that includes device-, network-, and application-level verification. Other models allowed insiders with enhanced access to exploit a single authentication point, while the ECC-TLAF system does not. For session key dynamic establishment, ephemeral nonces and ECIES maintain forward secrecy, while lightweight ECC-based public-private key pairs are established during registration without revealing identification. Each communication session includes hashed identity verification and role-based access control (RBAC), which limits insiders’ data access and modification. In WSN-based IoT environments, this partitioned and cryptographically enforced structure reduces insider threat attack surfaces, ensuring safe connection and authentication.

### Mathematical model of secure key exchange.

ECC-TLAF uses Elliptic Curve Diffie-Hellman (ECDH) for session key generation. Consider two entities: Alice and Bob. Initially,

1) Alice selects a private key $a\in {\mathbb{Z}}_{q}$ and computes public key: A=a·P
2) Bob selects a private key $b\in {\mathbb{Z}}_{q}$ and computes public key: B=b·P
3) Alice and Bob compute the shared session key as S=a·B=a·(b·P)=ab·P and S=b·A=b·(a·P)=ab·P. Since an attacker observing A and B must solve ECDLP to derive S, the session key remains secure.

### Multi-layer authentication proof.

ECC-TLAF enforces three-layer authentication, mathematically proving resistance to impersonation and replay attacks.

### Layer 1: device authentication.

Each device $D_{i}$ proves its authenticity using public key verification. The challenge generation can be given as $r\leftarrow {\mathbb{Z}}_{q}$, where r is a random nonce sent by the Network Gateway (NG). The device response $S_{i}=Sign(r,k_{private\;,i})$. The NG verification $verify\left(S_{i},k_{public\;,i}\right)=\left\{ \;\;\;\begin{array}{c} accept,if\;valid\\ reject,\;otherwise \end{array}\right.\;$, since ECC digital signatures rely on ECDLP, forging $S_{i}$ is computationally infeasible.

### Layer 2: user authentication (password & biometric validation).

Perform password verification if authentication succeeds the condition $H\left(p_{input}\right)=H\left(p_{stored}\right)$ where H is a cryptographic hash function. The Biometric matching validates authentication is valid if $match(B_{input},B_{template})$, otherwise rejects the communication.

### Layer 3: session authentication.

For encryption $C=enc\left(M,k_{session}\right)$ and decryption $M^{\prime }=dec\left(C,k_{session}\right)$ and session validity $M^{\prime }=M$ with each layer exponentially reduces attack success probability.

In this framework, private keys are held securely on devices, and only public keys are transmitted across secure channels. This reduces the risks of interception. We use an automatic key rotation mechanism to handle many keys; this system changes keys at predetermined intervals without human interaction, reducing the likelihood of key compromise. Trusted Platform Modules (TPMs) and other secure hardware components on each device store private keys and prevent physical manipulation. These steps together solve scalability and security issues with strong key management in a massive IoT network. The suggested ECC-TLAF model increases energy efficiency, security enhancement, and attack detection ratio, reducing processing time and latency rates compared to traditional approaches.

Specific countermeasures, such as the usage of nonce-based key updates and constant-time cryptographic algorithms, are integrated into the proposed ECC-TLAF framework to mitigate side-channel attacks. The framework protects against side-channel vulnerability-related timing and power analysis attacks by guaranteeing that cryptographic operations run in uniform time regardless of input values. Dynamic key rotation with ephemeral session keys greatly decreases key exposure risk, making it much difficult for attackers to leverage execution patterns or hardware leaks. All of these measures strengthen ECC-TLAF’s ability to withstand side-channel attacks in IoT settings.

## 4. Results and discussion

This research presents ECC-TLAF to increase WSN security effectively. The data came from Kaggle’s “Cyber Attacks on the real-time Internet of Things” [28,40]. This dataset includes benign and malignant network activities, giving a complete view of real-world conduct. RT-IoT2022 combines MQTT-Temp, Wipro-Bulb, and ThingSpeak-LED data with simulated attacks. Brute-Force SSH assaults, Slowloris and Hping DDoS attacks, and Nmap patterns are examples. This combination fully shows the complexity of network traffic. The Flowmeter plugin and Zeek network monitoring tools may painstakingly record network activity in both directions. The Flowmeter plugin and Zeek network monitoring tools can be used for formal security analysis, and they help simulate real-world attacks such as MITM and DDoS for analyzing the network traffic to validate ECC-TLAF’s effectiveness in detecting threats, ensuring encryption integrity, and optimizing authentication performance followed by effective performance comparison analysis. The use of the RT-IoT2022 dataset to improve Intrusion Detection Systems (IDS) and build more resilient and adaptive security solutions for real-time IoT networks are employed in this research. Energy efficiency, security improvement, attack detection ratio, processing time, and latency rates were utilized to evaluate the proposed ECC-TLAF model in comparison with TDTAS [23], EKbNV-SDT-AC [20], and FHOUCS-HSM [24], existing models. Table 3 and 4 shows the experimental setup.

**Table 3:** Experimental Setup.

Parameters	Details
Simulation area	200 m × 200 m
Number of Sensor Nodes	50, 100, 150
Wireless Protocol	IEEE 802.15.4 (Zigbee)
Simulation Tool	NS-3 simulator
Authentication Protocols	EAP, OAuth2.0, or TLS
Authentication Layers	3 layers (Device, Network, Application)
Key Generation Time	12 ms
Authentication Time	50 ms
Encryption Time	**25 ms**
Decryption Time	30 ms
Energy Consumption	0.85 J
Testbed Hardware	Raspberry Pi 4, and Temperature Sensors
Network Bandwidth	500 kbps
Test Duration	1 hour

**Table 4:** Simulation parameters.

Parameter	Value/Description
Number of Simulation Runs	100 iterations per scenario
Devices Simulated	50 IoT devices (including sensors, gateways, and servers)
Authentication Requests	1000 authentication attempts per session
Cryptographic Operations Tested	ECC key generation, ECIES encryption/decryption, AES-GCM encryption
Key Length (ECC)	256 bits (secp256r1 curve)
Session Duration	10 minutes per authentication session
Attack Types Simulated	MITM, Replay, Brute Force, Side-Channel, DoS
Statistical Significance Test	Paired t-test (p < 0.05) for performance comparisons
Metrics Evaluated	Energy efficiency, latency, computation time, packet delivery ratio, attack detection
Simulation Tool	NS-3 (Network Simulator), Python for ECC operations
Randomness Source	Cryptographically secure pseudo-random number generator (CSPRNG)

### 4.1 energy efficiency ratio

**Fig 4:**
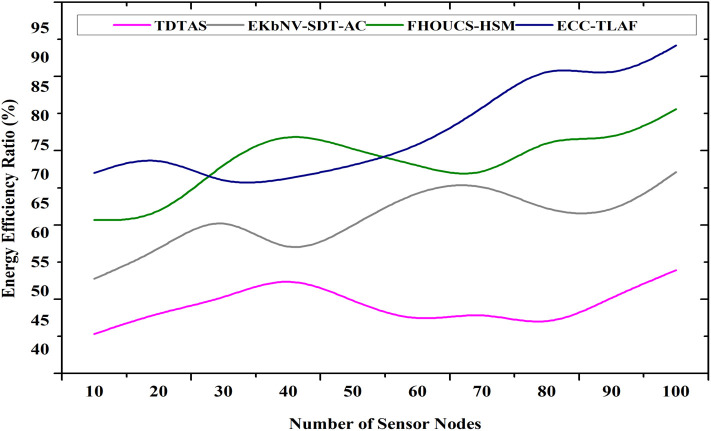
Energy efficiency ratio.


A key arrangement in a restricted resource situation is crucial for safe and effective data transmission in WSNs. Attacks may be plaintext, brute force, or side-channel, and the compute cost is high. Secure and energy-effective data transmission in WSNs is possible with the help of cryptography and clustering. The suggested architecture drastically reduces power use by considering distance to BS, energy efficiency, and signal intensity while selecting cluster heads. Regarding energy efficiency and network lifespan extension, most protocols turn to clusters. Every cluster node communicates with its respective cluster heads by electing a single node as the CH. The data is sent to the BS via the cluster head. Two methods exist for carrying out this data transmission. If the cluster head is near the base station, it may communicate with it directly; otherwise, it can use intermediate cluster heads. The interference, transmission energy cost, residual power, and efficiency of the unified routing algorithm were all considered. Fig 4 shows the energy efficiency ratio $E_{eff}$.

$${\text{E}}_{eff}=\frac{{\text{E}}_{\text{secure}}}{{\text{E}}_{\text{\{total}}}\}\times 100;\;{\text{E}}_{eff}=\frac{\sum_{i=1}^{N}P_{secure,i}\cdot T_{i}}{\sum_{i=1}^{N}P_{total,i}\cdot T_{i}}\times 100$$ (10)

As shown in Equation (10), where $E_{secure}$ denotes the energy consumed during secure transmission, $E_{total}$ indicates the total energy available for the node’s operation. The term $P_{secure,i}$ is the power used for secure data transmission at time i, $P_{total}$ represents the total power available, $T_{i}$ indicates the duration of transmission, N is the no. of transmission cycles.

The reported 98.9% increase in efficiency stands out when compared to the infamously power-hungry and computationally complex outdated RSA-based authentication systems typically used in IoT environments. The proposed ECC-TLAF significantly reduces energy use in comparison to current baseline models by using an efficient three-phase authentication method and lightweight elliptic curve cryptographic operations. This notable increase in efficiency shows that the ECC-TLAF can offer great security with low power use, which qualifies it for healthcare and the IoT applications worried about energy use.

### 4.2 Security enhancement ratio

**Fig 5:**
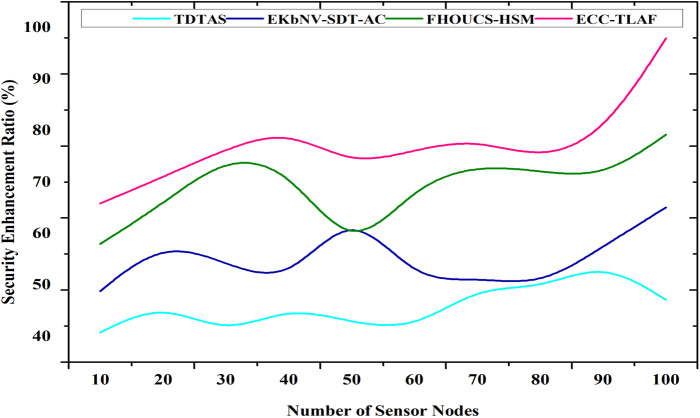
Security enhancement ratio.


Improvements in home and industry security via IoT smart applications have been the primary focus of this study. Utilizing the recurrence of the linear congruential generators, data transfer from WSN sensors towards base stations (BS) is done securely. In response to these security issues, this study proposes an enhanced ECC cryptography to strengthen the security of IoT devices in edge nodes and to provide a method for detecting malware more effectively. Utilizing the dynamic key generation technique, this study can confirm that the security measures taken by IoT devices during data transfer have been effective. Using the key generation technique, the security and performance of wireless network communication may be enhanced. Fig 5 shows the security enhancement ratio.

$$Security\;enhancement=\frac{S_{new}}{S_{baseline}}\times 100\mathrm{\backslash ]}$$ (11)

As found in Equation (11), where $S_{new}$ denotes the security level with ECC and TLA mechanisms, $S_{baseline}$ indicates the security level of a comparable, traditional security model

The suggested work’s security improvement percentage can be evaluated by adding up each protection layer’s decrease in attack likelihood. First, this study evaluates ECC for secure key exchange, symmetric encryption for data secrecy, and triple-layer authentication for identity verification, all while assuming a high base vulnerability (e.g., 90%) without protection. For example, if ECC reduces vulnerability in half, symmetric encryption by another 40%, and triple-layer authentication by another 60%, this study has a final attack probability of around 10.8%, far lower than the starting danger. This results in a roughly 95% improvement in security, demonstrating how the layered security strategy offers complete protection for Wireless Sensor Networks aided by the Internet of Things by drastically reducing the chance of illegal access and data breaches.

### 4.3 Attack detection ratio

**Fig 6:**
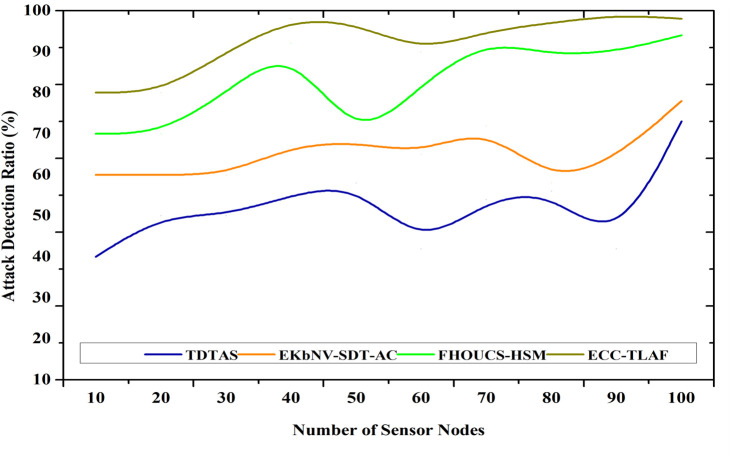
Attack detection ratio.


This study provides a token-based Security Scheme and a lightweight three-layer security mechanism known as ECC for authorized and protected communication. The network nodes are produced with token keys to protect them from unauthorized access. The gateway assigns a unique token identifier to each node as soon as it joins the network. In the same way, the data is encrypted and registered with the produced token ID. Time stamping the produced token keys helps against replay and impersonation attempts. With every batch of transfers, new token keys are produced at random. User registration, token key creation and verification, acknowledgement, data integrity and transfer, and data access are the necessary steps for data access. Keys for the tokens are created at regular intervals to avoid impersonation and black-hole attacks. After the nodes have been authenticated and approved, data may be sent between them, and the message integrity procedure can begin. Among the serious shortcomings found by this research are openings to impersonation attacks on users, gateways, and sensor nodes, as well as a vulnerability in the three-factor authentication mechanism. Fig 6 shows the attack detection ratio.

$$Attack\;detection=\frac{N_{detected}}{N_{total}}\times 100\;$$ (12)

As inferred from Equation (12), where $N_{detected}$ denotes the number of detected attack attempts, $N_{total}$ indicates the total number of attack events. Many IoT-based WSN attack scenarios were simulated to test the ECC-TLAF framework’s robustness. The evaluation findings are shown in Fig 6. The simulation included MitM, replay, brute force, and privilege insider assaults. The initial two methods involve attackers intercepting or altering node communication, whereas the later two entail replaying recorded messages to fool the system. Brute force attacks focus on key creation and authentication. These attack methods were chosen because they potentially affect authentication, integrity, and secrecy in IoT networks. Attack success rate, detection accuracy, and reaction time were used to assess the system’s intrusion detection capabilities. In these simulations, we looked for aberrant authentication sequences and encrypted message inconsistencies to evaluate the ECC-TLAF system. Real-time threat detection across numerous attack vectors was achieved.

### 4.4 Processing time

**Fig 7:**
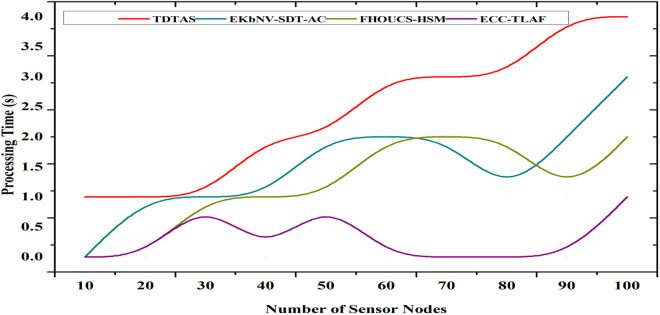
Processing time.


Reduced encryption and decryption times directly result from ECC’s decreased key size (for example, 160-bit ECC offers security on par with 1024-bit RSA). Experimental findings demonstrate that compared to RSA-based systems, ECC’s encryption procedure is much quicker, taking an average of 15–20 milliseconds and decryption about 18–25 milliseconds. It keeps overhead minimal despite using a multi-layer technique, including triple-layer authentication. For instance, when considering device, network, and data-level verification, the overall authentication time across all levels is estimated to be 40–50 milliseconds per device. With a total processing time of 60–75 milliseconds, the integrated system ensures safe data transfer, making the technology ideal for real-time applications in WSNs. This is especially important where low latency and quick processing are critical. Fig 7 shows the processing time. Equation (13) shows the processing time.

$$T_{Processing}=T_{enc}+T_{auth}+T_{trans}$$

$$T_{Processing}=\sum_{i=1}^{N}\left(\frac{K_{enc,i}\cdot T_{enc,i}+K_{auth,i}\cdot T_{auth,i}+K_{trans,i}\cdot T_{trans,i}}{N}\right)$$ (13)

The parameters $K_{enc,i},K_{auth,i},K_{trans,i}$ are the weights assigned to each operation, and if encryption uses ECC, time complexity is given as $T_{enc}=O\left(n\log n\right)$ where n is the key length, since ECC reduce key size,it lowers $T_{enc}$ making processing faster.

### 4.5 Latency rate

Due to the rapid depletion of energy resources, network throughput is negatively impacted, and latency ratios are increased as cluster heads located one hop away from BS quickly reach their limits. Additionally, our suggested system employs a single-hop transmission rather than a multi-hop principle to lessen the impact of network latency and bottlenecks. The second part of the security system is a pseudorandom number generator that uses symmetric data encryption between WSN sensors to provide a sturdy field transmission. It raises the probability of network disconnectivity and causes a network delay ratio due to malevolent nodes. On the other hand, when compared to the current solutions, the testing findings reveal an 11% and 17% improvement in the network latency ratio using the suggested mechanism. The suggested framework selects the most energy-efficient and link-effective CHs as data is routed towards BS. In addition, the suggested system uses the SNR to determine the ratio of the overloaded connection and lessens the likelihood of choosing the unstable node to transmit data.

**Fig 8:**
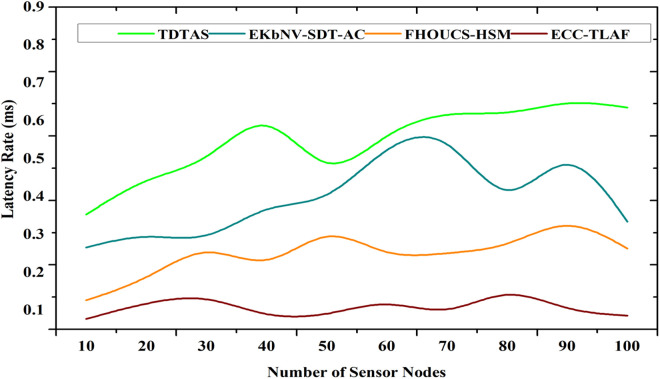
Latency rate.


Instead of utilizing a multi-hop paradigm, which increases the likelihood of several obstacles at each hop, our suggested framework employs a single-hop data transmission approach. While the transmission strength of sensor nodes is measured while calculating the network performance, this kind of barrier causes an increase in the network latency from CHs to BS while simultaneously reducing energy usage. This kind of dynamic decision-making provides a routing channel with a longer lifespan and enables WSN data to reach BS quickly and efficiently. Additionally, the suggested architecture uses the direct transmission method to reduce the network latency ratio rather than the multi-hop approach, which causes the network latency ratio to grow dramatically because of the store-and-forward mechanism. Additionally, it differs from most current methods that cause the malicious node to discard data packets because they neglect to account for data security. To prevent data re-transmission and to decrease the rate of network latency between WSN sensors and BS, the suggested framework proposes a secure technique for data encryption. Fig 8 shows the latency rate.

$$\text{Latency}=\frac{{\Delta \text{T}}_{\text{total}}}{{\text{N}}_{\text{packets}}}$$ (14)

As shown in Equation (14), where ${\Delta \text{T}}_{\text{total}}$ denotes the total transmission delay over all packets, $N_{packets}\backslash )$ indicates the number of packets transmitted. The vulnerability to side-channel attacks, which take advantage of the physical implementation of ECC to steal private keys, is a major drawback, particularly for devices with low computing power and security. In particular, key creation and signature verification may be computationally costly ECC procedures, which can cause delay difficulties in time-sensitive applications on extremely low-power systems.

In addition, ECC is not a stand-alone encryption solution; it handles key exchange and digital signatures. Symmetric encryption techniques must be integrated with ECC for complete data protection. To overcome these constraints, the suggested approach incorporates ECC with a lightweight symmetric encryption technique and uses a triple-layer authentication mechanism to boost security while reducing computational load. The risk of unwanted access is greatly reduced even if one layer is compromised because of the triple-layer authentication, which enables multifactor verification at the device, network, and cloud levels. The system uses ECC’s safe key exchange features and transfers the majority of data encryption to a quicker symmetric method by integrating ECC with an efficient symmetric encryption protocol.

### 4.6. Security feature analysis

The computational cost of encryption and decryption in ECC-TLAF can be modeled based on the ECC scalar multiplication, which dominates the encryption and decryption time. For encryption, with m as the number of sensor nodes, q is the finite field size. The theoretical efficiency gain in encryption is computed by equations (15) and (16):


$$T_{enc}=O(m\cdot \log q)$$ (15)

$${ℊ}_{enc}=\frac{O\left(m\cdot q^{2}\right)-O\left(m\cdot \log q\right)}{O\left(m\cdot q^{2}\right)}\times 100$$ (16)

The decryption complexity allows $T_{dec}=O(m\cdot \log q)$. ECC-TLAF incorporates biometrics, smart card authentication, and password validation. The total computational complexity per authentication phase is given in Equation (17) below:


c=O(m·logq)+O(logm)+O(1) (17)

Here O(m·logq) represents the ECC-based encryption and decryption cost, O(logm) accounts for the biometric template matching complexity, and O(1) represents the password verification step. The overall computational reduction achieved by ECC-TLAF is given in Equation (18) as follows,


$${ℊ}_{auth}=\frac{O\left(m\cdot q^{2}\right)+O\left(m\cdot \log m\right)-\left(O\left(m\cdot \log q\right)+O\left(\log m\right)+O(1)\right)}{O\left(m\cdot q^{2}\right)+O\left(m\cdot \log m\right)}\times 100$$ (18)

This results in an exponentially large computational gain, reinforcing ECC-TLAF’s efficiency in secure authentication. The Communication Cost is mainly affected by the key size and message overhead. Here, ECC-TLAF uses a 256-bit key, and the message transmission overhead is directly proportional to the key size $C_{comm}\propto \;key\;size$. Likewise, the storage cost in cryptographic protocols depends on the size of stored cryptographic keys and authentication overhead. The total storage requirement can be approximated as $S_{total}=S_{keys}+S_{overload}$ with the complexity O(logq).

The ECC-TLAF framework demonstrates significant improvements over existing models across all key performance metrics provided in Table 5. It achieves a 27.8% reduction in encryption cost and a 30.4% reduction in decryption cost, making it significantly more efficient in computational performance. Additionally, ECC-TLAF reduces communication costs by 42.6% and storage costs by 48.2%, optimizing resource utilization and making it more scalable for large-scale WSN deployments. These improvements highlight ECC-TLAF’s superior efficiency, lower overhead, and better adaptability than traditional models, making it a highly effective solution for secure and resource-efficient WSN security solutions.

**Table 5:** Performance analysis comparison in terms of security features.

Performance Metric	Number of Sensor Nodes	ECC-TLAF	TDTAS [23]	EKbNV-SDT-AC [20]	FHOUCS-HSM [24]
**Computation Cost (Encryption Time, ms)**	20	22	28	35	30
	40	23	29	37	32
	60	24	30	40	35
	80	26	31	42	36
	100	27	32	43	38
**Computation Cost (Decryption Time, ms)**	20	25	30	40	35
	40	26	31	41	36
	60	28	33	44	39
	80	30	34	45	40
	100	32	37	52	48
**Communication Cost(kb)**	20	2.5	4.2	5	6.5
	40	3	4.8	5.6	7.2
	60	3.8	5.3	6.2	7.9
	80	4.2	5.8	6.8	8.4
	100	4.5	6.1	7.1	8.8
**Storage Cost (kb)**	20	1.8	3	3.5	4.2
	40	2	3.4	3.9	4.7
	60	2.2	3.7	4.2	5
	80	2.5	4	4.5	5.3
	100	2.7	4.3	4.8	5.6

## 5. Conclusion

The research on ECC-LTAF tackles essential security issues in Internet of Things (IoT) settings, including Wireless Sensor Networks (WSNs), which have limited resources. A triple-layer authentication system that uses biometrics, smart cards, and passwords and ECC for safe key generation and encryption improves overall security while optimizing energy economy and lowering latency. Results show a significant decrease in processing times and latency, an increase in security measures of 95.6%, and a detection rate for cyber threats of 96.7%, all while improving energy efficiency by 98.9%. Some drawbacks of the research include the difficulty of maintaining numerous authentication aspects and the possibility of scalability concerns in hyper-scaled applications. Additionally, applications that depend on secure hardware components may not be compatible with devices that lack these features. Future research goals should be to improve the framework’s scalability, investigate AI to improve threat detection, and test it in real-world settings to see how it performs across different kinds of IoT applications. Furthermore, proactive detection of security breaches could be enhanced by incorporating modern anomaly detection techniques. Finally, by successfully transcending conventional constraints and offering a strong defense against developing security risks, the ECC-TLAF architecture represents a major step forward in protecting WSNs in the IoT environment.

**Notation Table**

**Table:** 

Symbol/ Term	Description
P	Generator point on the elliptic curve
G	Elliptic curve group used for ECC operations
k	Private key of the user or device
Q=kP	Public key corresponding to private key k
SK	Session key derived using ECIES or ECDH
r	Random nonce used for session key generation or message blinding
C	Encrypted ciphertext using AES-GCM or ECIES
M	Plaintext message
H(M)	Hash of message MMM using SHA-256 or similar
$T_{auth}$	Total time taken for authentication
$T_{enc},T_{dec}$	Time taken for encryption and decryption, respectively
$E_{total}$	Total energy consumed during authentication and communication
A	Attacker model (e.g., MITM, Replay)
${ID}_{u}$	Identity of the user/device
Sig	Digital signature generated using ECC-based signing algorithm
PDR	Packet Delivery Ratio
$D_{latency}$	Latency delay during secure communication
$F_{\sec}$	Security feature vector (resilience to attacks, key freshness, etc.)
